# The Effect of Irradiation on Mechanical and Thermal Properties of Selected Types of Polymers

**DOI:** 10.3390/polym10020158

**Published:** 2018-02-07

**Authors:** David Manas, Martin Ovsik, Ales Mizera, Miroslav Manas, Lenka Hylova, Martin Bednarik, Michal Stanek

**Affiliations:** 1 Tomas Bata University in Zlin, Faculty of Technology, Vavreckova 275, 760 01 Zlín, Czech Republic; dmanas@utb.cz (D.M.); hylova@utb.cz (L.H.); mbednarik@utb.cz (M.B.); stanek@utb.cz (M.S.); 2 Tomas Bata University in Zlin, Faculty of Applied Informatics, CEBIA-Tech, Nad Stranemi 4511, 760 05 Zlin, Czech Republic; mizera@utb.cz (A.M.); manas@utb.cz (M.M.)

**Keywords:** crosslinking, beta rays, micro-indentation, TMA (thermo-mechanical analysis), X-ray, gel content

## Abstract

This article deals with the influence of electron-beam radiation on the micro-mechanical, thermo-mechanical, and structural properties of selected polymers. In the search for the desired improvement of polymers, it is possible to use, inter alia, one particular possible modification—Namely, crosslinking—Which is a process during which macromolecular chains start to connect to each other and, thus, create the spatial network in the structure. In the course of the treatment of the ionizing radiation, two actions can occur: crosslinking and scission of macromolecules, or degradation. Both these processes run in parallel. Using the crosslinking technology, standard and technical polymers can acquire the more “expensive” high-tech polymeric material properties and, thus, replace these materials in many applications. The polymers that were tested were selected from across the whole spectra of thermoplastics, ranging from commodity polymers, technical polymers, as well as high-performance polymers. These polymers were irradiated by different doses of beta radiation (33, 66, 99, 132, 165, and 198 kGy). The micro-mechanical and thermo-mechanical properties of these polymers were measured. When considering the results, it is obvious that irradiation acts on each polymer differently but, always when the optimal dose was found, the mechanical properties increased by up to 36%. The changes of micro-mechanical and thermo-mechanical properties were confirmed by structural measurement when the change of the micro-hardness and modulus corresponded to the crystalline phase change as determined by X-ray and gel content.

## 1. Introduction

Material modification is needed in order to obtain better material properties as required in the plastics industry. One of many ways of how to modify polymers is beta radiation crosslinking. In most cases, crosslinked polymers offer better mechanical and thermal properties. The beta radiation sources for the industrial crosslinking of polymers are electron beam accelerators, which allow one to obtain high-radiation doses in a short time [1]. Ionizing radiation (electron beam radiation) can change the macroscopic properties and the molecular structure of polymeric materials. Thermoplastic materials demonstrate better resistance to temperature-induced deformation or flow, and do not melt after the creation of crosslinking bonds. After crosslinking, the impact resistance, chemical resistance, toughness, and thermal stability are all improved [2]. This study focuses on the electron beam crosslinking of various types of polymers. Herein, the individual polymers are described, as are the studies already carried out on their crosslinking by electron beam radiation.

The first polymer used in our study is polyethylene. Polyethylenes are commodity polymers, which account for more than 70% of total plastics consumption, and are low-cost, easily processable, and available. Typical applications of these polymers include packaging, household items, net ropes, medical applications, fishing rods, water pipes, etc. There are many types of polyethylenes, including linear low-density polyethylene, low-density polyethylene, ethyl vinyl acetate copolymer, polyolefin elastomer, high-density polyethylene, and many others [3]. After the addition of crosslinks in low-density polyethylene (LDPE), the material displays ductile plastic behavior below its melting range. After the melting of all crystallites, the behavior of LDPE becomes elastomeric. Crosslinked LDPEs (XLDPEs) have found to have a wide range of applications in the production field—Films, sheets, and foams [4]. Nowadays, LDPE has, in most cases, been replaced by XLDPE. The heat deformation behavior of XLDPE is superior in comparison to LDPE. The crosslinking of LDPE can be initiated by using peroxide, introducing silane groups into polyethylene, or by radiation; e.g., electron beam or gamma ray radiation [5].

High-density polyethylene (HDPE), was used as a second commodity polymer, it is a semi-crystalline thermoplastic material belonging to the polyolefin family. Mehrjerdi et al. [6] studied HDPE, containing 2.5 wt % of carbon black (CB), and talc—Fully blended by the supplier. They realized that CB proved to be an effective additive for the improvement of thermal stability, while it had a poor influence on mechanical properties, especially on impact resistance. In the case of the influence of talc on material properties, the thermal diffusivity and conductivity, and the specific density and the toughness perpendicular to the direction of flow, were improved, while the specific heat capacity of the composites decreased [6]. Another investigation concentrated on HDPE, which focused on critical considerations for the accelerated ageing of high-density polyethylene potable water materials; this was the first investigation to demonstrate water sorption and desorption by HDPE resin and HDPE potable water pipes. The scientists they determined the recommended water quality conditions for the accelerated ageing of polyethylene materials, which they then inserted into tables [7].

The last polyolefinic polymer used in our investigation is polypropylene (PP), which ranks among the most consumed polymers globally, is a non-polar thermoplastic semi-crystalline polymer. It is a material that is non-hazardous and non-toxic, which is why it is widely used in the plastics industry. PP demonstrates good resistance to alkalis and acids, electrical insulation and processing, bending fatigue resistance, and its chemical stability is also great; unlike PPs, its mechanical properties are low; however, its mechanical properties can be improved by reinforcing with fillers [8,9]. Sombatsompop and Chaiwattanpipat [10] investigated the difference between the injection molding of pure polypropylene and polypropylene with different contents of glass fibers. They found that the melt temperature is influenced by the presence of glass fibers due to shear heating between polymer-polymer and polymer-glass fibers during the melt flow. The increase in the melt temperature during the flow appears to be more significant with the reinforcement of glass fibers due to the shear heating in comparison with pure PP [10]. Köpplmayr et al. [11] dealt with the fiber orientation and length distribution and the rheological characterization of glass fiber-reinforced PP. They prepared three types of blends: compounds with 100% short fibers, 20% long, and 80% short fibers, and 70% long, and 30% short fibers, the wt % of glass fibers in all three compounds was 24 wt %. Their study´s results showed the different rheological behavior depended on fiber content and orientation as well as elongation and shear viscosities, etc. [11].

As the fourth polymer for our study, we selected polybuthylene terephthalate (PBT), a semi-crystalline thermoplastic polymer that belongs to the polyester family, is characterized by its high rigidity, low tendency to creep, high strength, dimensional stability, good impact strength, very low thermal expansion coefficient, good wear and frictional resistance, good chemical resistance to acids, and very low moisture absorption, etc. PBT is mainly used in the automotive, consumer applications, electric, and textile industries and is very often modified with particulate fillers, like other polymers [12,13]. It was found that beta radiation crosslinking can significantly improve the mechanical properties, such as micro-hardness, indentation modulus, and indentation creep. The most important aspect is to choose the appropriate dose of beta radiation which, in the case of indentation modulus improvement, is 132 and 66 kGy in the case of micro-hardness improvement. This depends on which property is required, how to improve it, and for which applications [13,14].

Polyamides (PA) very often find their use in industrial practice. Two types of polyamides were used, namely, Nylon 6 (PA6) and polyamide 9T (PA9T). Nylon 6 is a semi-crystalline aliphatic polyamide, whose advantages are low friction coefficient, high fatigue strength, and high resistance to a wide range of chemicals, oils, and fuels. It is an important engineering polymer—Used in a widespread spectrum of applications—For example, the automotive, construction, transportation, and other industries, [15]. The scientists Dadbin, Frounchi, and Goudarzi [16] dealt with electron beam-crosslinked nylon 6, and changes in the absorbed dosage dependency on its properties. As a crosslinking agent, researchers used TAC (triallyl cyanurate) and, for crosslinking purposes, they used an electron accelerator with an energy level of 5 MeV, under irradiation doses from 40 to 150 kGy. These confirmed that various amounts of crosslinking agent had various influences on polymer properties. The higher the absorbed dose of beta radiation and the amount of TAC, the lower the water absorption of polyamide 6. Crystallinity, in a very similar way, decreased to 1.2% and 3% of TAC, in line with the increasing absorbed dose, in comparison with virgin nylon 6 without TAC. The melting temperature was the lowest at a dose of 80 kGy, amounting to 3% TAC, which can be positively used in working practice to lower cost production [16]. In order to obtain even better mechanical properties, it is possible to reinforce a polyamide by using glass fiber. Glass fiber-reinforced polymer (GFRP) represents a conformable design solution due to its special production adaptability, structural efficiency, and high durability. Its usage is also advantageous due to increasingly low production and erection costs [17]. Porubska et al. [18] examined the effect of electron beam irradiation on the properties of virgin and glass fiber-reinforced polyamide 6. They determined that crosslinking is more beneficial for virgin polyamide because of the increase in tensile strength at break and Young’s modulus. Properties at yield were not influenced by crosslinking, thermal resistance was only marginally influenced by irradiation for both GFRD (a marginal decrease), and for virgin polyamide, a small increase. Their finding is that electron beam irradiation leads to more changes in the properties of virgin polyamide in comparison with GFRD [18].

Polyamide 9T (PA9T) is a new, semi-aromatic, high-performance, engineering polyamide. This polymer has a long, flexible aliphatic linkage that consists of nine methylene groups in a sequence included in the main aromatic polymer chain. This polymer has a good balance of manufacturing cost and properties, and has good heat stability, low water absorption, considerable resistance to hot water, acid, alkali and organic solvents, and high heat moisture resistance. Due to these properties, PA9T is used in electrical, electronic, and automobile parts. Due to production factors, its fiber is also now used for the production of fishing nets and other industrial and textile materials [19].

Generally speaking, various crosslinking methods are used—Chemical crosslinking, silane crosslinking, peroxide crosslinking UV crosslinking, and irradiation crosslinking—All of which cover crosslinking via accelerating electron beams or gamma rays. For the production of technical components/parts foils, cable electric insulation, pipes, and houses, etc., electron beam irradiation leading to polymer crosslinking has many advantages in comparison to other methods. First of all, the processing condition in the production of plastic parts (injection molding, extrusion etc.), is not influenced by crosslinking, since crosslinking itself is realized after processing. The crosslinking process is very rapid and can be realized in a simple way. Due to the possibility of changing the degree of crosslinking by means of the irradiation dose, it is also possible to modify/tailor polymers to customers’ needs. Electron beam crosslinking is considered to be the cleanest—And most environmentally-friendly—Crosslinking method since it does not involve other chemicals and uses only high energy electrons to perform. A possible disadvantage of irradiation crosslinking could be seen to lie in the need for significant investment and high requirements of operation safety zones.

Polymer behavior after irradiation depends on the polymer type. The effectiveness of irradiation depends on many factors that affect the structural changes of the irradiated polymers, and the resulting properties, both quantitatively and qualitatively. The main material and process factors—e.g., physical aspect (i.e., *T*_g_, crystallinity), chemical structure, molecular weight and molecular weight distribution, polymer configuration, irradiation dose, irradiation source intensity, environmental parameters (e.g., air, nitrogen, vacuum), environmental temperature, as well as parameters, like the geometry of plastic products (thickness)—Can effectively be influenced by the addition of crosslinking agents: for example, TAC (triallyl cyanurate), TAIC (triallyl isocyanurate), and many others [20].

Two main processes occurred during the polymer irradiation crosslinking and degradation. Mainly, the chemical structure of the polymer effects the proportion of crosslinking and degradation. It is possible to differentiate polymers into two groups. Polymers from the first group (polyethylene, polystyrene, etc.)—Predominantly crosslink, their macromolecular chains, connected in 3D structures with high molecular weight, became lower—And the molecular chains are scissioned by (polypropylene, polymethylmetacrylate etc.), [21]. These problems were studied by Miller [22], Maklis [23], and Drobny [24].

**Figure 1:**
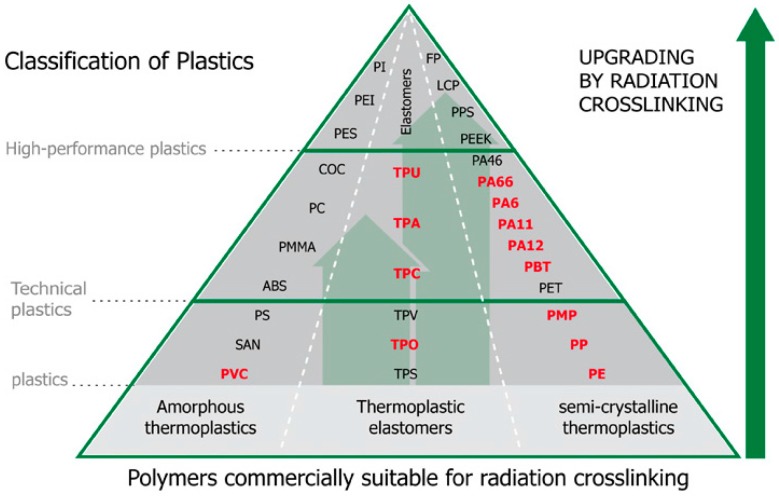
Upgrading properties by radiation crosslinking [25].

The thermoplastics that are used for the production of various types of products have very different properties. Standard polymers are easily obtainable with favorable price conditions are ranked among the main class. The use of standard polymers is limited, both by mechanical and thermal properties (Figure 1). The group of standard polymers is the one worth the most consideration, and its share in the production of all polymers is as high as 90%.

This study deals with the influence of electron beam radiation on selected types of thermoplastics selected across the whole spectra of polymers (i.e., commodity, technical, and high-performance polymers). The micro-mechanical and thermo-mechanical properties of the tested polymers were measured, and these demonstrated an increase in the described properties. These changes were confirmed by structural measurement (Raman, X-ray, gel content).

## 2. Materials and Methods

Crosslinked materials were chosen for the measurement of their micro-mechanical and thermo-mechanical properties for comparison purposes of various options. The test samples were made from this material, according to the standard ISO 527-2 1BA for the tensile test. The injection molding and radiation crosslinking processes were performed with a minimum time gap to avoid its influence on the measurement surroundings.

### 2.1. Material

The polymers tested were selected across the whole area of thermoplastics regarding commodity, technical, and high-performance polymers (Table 1):

- Commodity polymers—Low density polyethylene (LDPE), and high density polyethylene (HDPE), without a crosslinking agent.
- Technical polymers—Polypropylene, with 30% glass fibers (PP 30% GF); polybuthylene terephthalate (PBT), polyamide 6 (PA6), polyamide 6 with 30% glass fiber content (PA6 30% GF). The material already contained the special crosslinking agent TAIC (triallyl isocyanurate, 6 vol %), which should enable the subsequent crosslinking by means of ionizing β radiation.
- High-performance polymers—Polyamide 9T (PA9T). This material already contained the special crosslinking agent TAIC (triallyl isocyanurate, 6 vol %), which should enable subsequent crosslinking by ionizing β radiation.

### 2.2. Sample Preparation

The samples were made using injection molding technology on an Arburg Allrounder 470H injection molding machine (Loßburg, Germany). The normalized specimens, with dimensions of (80 × 10 × 4) mm, were used. The process parameters were set according to the manufacturer’s recommendations; see Table 2.

### 2.3. Irradiation

The irradiation of the tested polymers was performed with the kind assistance of BGS Germany (Wiehl, Germany), in the BGS Wiehl plant, using accelerated electrons. A RHODOTRON E-beam accelerator (Tongeren, Belgium) with 10 MeV. Electron energy was used for this purpose. The irradiation process of the specimens was performed under general conditions (air atmosphere, ambient temperature of 23 °C) just as it is done in engineering practice. The range of the doses was determined on the basis of experience gained from industrial irradiation practice, in the range of 33 to 198 kGy. Each passage under the accelerator scanner is equal to 33 kGy. The required dose was determined according to accelerator parameters and its correctness was measured by a dosimeter. A nylon FWT 60-00 dosimeter (Goleta, CA, USA) was used to check the correct radiation dose, followed by analysis carried out on a Genesis 5, spectrophotometer (Madison, WI, USA), in line with the ASTM 51261 standard.

### 2.4. Micro-Indentation

**Figure 2:**
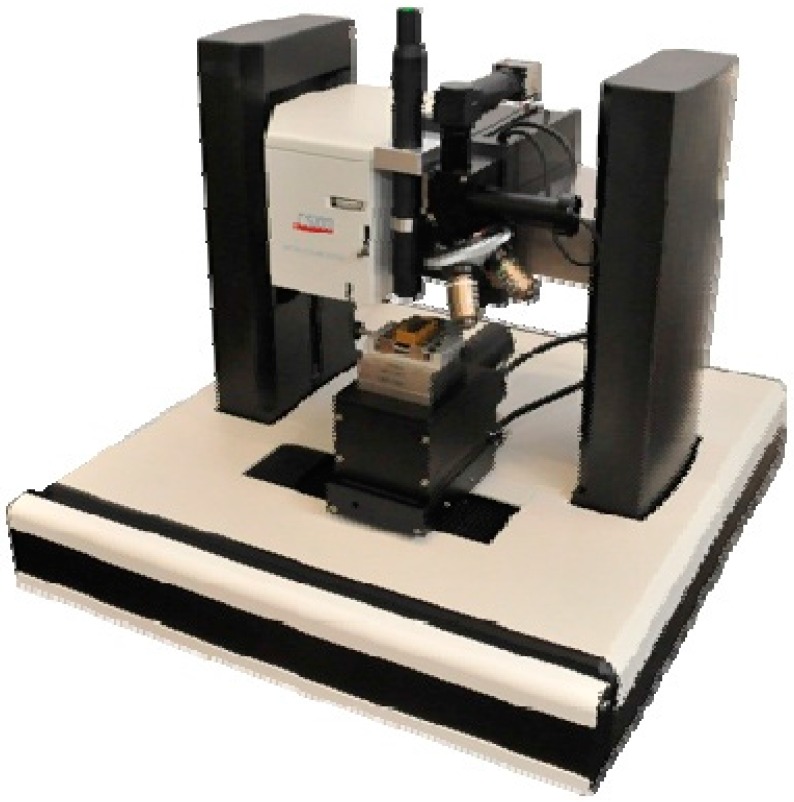
A micro-indentation tester.

Micro-indentation tests were performed using a micro-indentation tester (Micro Combi Tester) (Figure 2), made by Anton Paar (Graz, Austria), according to the CSN EN ISO 14577 standard. The tip is made of diamond and its shape is that of a cube corner (Vickers, Anton Paar, Graz, Austria). In the present study, the maximum load used was 1 N, and the loading rate (and unloading rate) was 2 N/min. The holding time was 90 s. The measurement was carried out using the depth sensing indentation (DSI) method. This method enables one to measure the force acting on the indentor, as well as the displacement of the indentor´s tip.

The indentation hardness (*H*_IT_), was calculated as the maximum load (*F*_max_), on the projected area of the hardness impression (*A*_p_); and the indentation modulus (*E*_IT_), is calculated from the plane strain modulus (E*), using an estimated sample Poisson’s ratio (*ν*):

(1) $$H_{\mathrm{IT}}=\frac{F_{\max}}{A_{p}}$$

(2) $$E_{\mathrm{IT}}=E*\cdot (1-v_{s}{}^{2})$$

Measurements of all of the above-mentioned properties were performed 10 times in order to ensure statistical correctness.

### 2.5. Gel Content

A gel test is condcuted to determine the content of non-filtered phase-gel of the given material according to the CSN EN 579 standard. The portion of 1 g (of material radiated by radiation doses), weighed with a precision of three decimal places, was mixed with 100–250 mL of solvent. Xylol was used for testing the polymers because it dissolves the amorphous part of polymers; the crosslinking part does not dissolve. The mixture was extracted for 6 h. Then, the solutes were separated by distillation. After removing the residual xylol, the crosslinked extract was rinsed in distilled water. The rinsed extract was then dried for 8 h, under vacuum, at 100 °C. The dried and cooled residue was re-weighed, with a precision of three decimal places, and compared to the original weight of the portion. The result, stated in percentage, shows the degree of crosslinking:

(3) $$G_{i}=\frac{m_{3}-m_{1}}{m_{2}-m_{1}}\;100$$

where *G_i_* is the degree of crosslinking of each specimen expressed in percentage, *m*_1_ is the weight of the cage and lid in milligrams; *m*_2_ is the total weight of the original specimen, cage, and lid in milligrams; and *m*_3_ is the total of the weight of the residue of specimen, cage and lid in milligrams.

### 2.6. X-ray Diffraction

X-ray diffraction patterns were determined using a PAN Analytical X-pert Prof X-ray diffraction system (Panalytical, Almelo, The Netherlands). The CuKα radiation was Ni-filtered. The scans (4.5° 2 Θ/min), in the reflection mode were taken in the range of 5–30° 2 Θ.

Each crystalline peak and amorphous halo has an individual Gauss function. Finally, the crystallinity of the samples was calculated as the ratio between the sum of the crystalline peak areas and the area of the whole X-ray pattern.

### 2.7. Raman Spectroscopy

Raman spectra were measured using an InVia Basis Raman microscope, made by the Renishaw Company, Gloucestershire, UK. This device is equipped with a Leica DM 2500 confocal microscope, Boston, MA, USA, with resolution up to 2 m. A laser with a wavelength of 514 nm and output power of 20 mW was chosen as the excitation source. The samples were measured at the power of 0.5 mW, the measured time was 10 s, and there were 5 accumulations of the spectrum. For micro-measurement purposes, a microscope lens with a magnification capacity of 50× was used. The measuring track was approximately 2 m. The samples were measured in the range of 500–1600 cm^−1^.

### 2.8. Thermo-Mechanical Analysis

Temperature stability was assessed using a thermo-mechanical analysis in the penetration mode. The thermo-mechanical properties were measured using a Perkin-ElmerDMA 7e thermal analyzer, Waltham, MA, USA, which was used for the thermo-mechanical analysis, heated from 50 to 400 °C (depending on the material used), at 20 °C/min, and held for 1 min at 50 °C. This precise temperature resistance evaluation of the polymers (e.g., irradiated, crosslinked polymers) records the displacement of the probe with a loading of 160 mN, which penetrates into the heated material, in a set range of temperatures.

### 2.9. Visual Observation

Visual observation was carried out in a temperature chamber at different temperatures for each material used, with a holding time of one hour on the temperature used and, after this time, photographs were taken. This technique is not as precise or as sensitive as a TMA but, from the macroscopic point of view, deformation under temperature influences is visible at first sight.

The test bodies were loaded in the horizontal position, with a bending moment from their own weight and the bending moment from the force acting on the body-end (1.5 N·mm).

## 3. Results

Suitable materials were selected for radiation crosslinking for micro-mechanical and thermo-mechanical property measurement purposes. Three groups of materials were chosen (commodity polymers, technical polymers, and high-performance polymers). The main reason for the selection of these materials from these groups is their easy modification by beta radiation, and their frequent application in technical practice. Structural measurements were performed so as to confirm the results and changes of the micro-mechanical and thermo-mechanical properties of the tested polymers. The aim of these measurements being to describe changes in structure and morphology in the polymers tested by irradiated beta radiation. These properties were always measured 10 times for all of the tested materials.

### 3.1. Commodity Polymers

#### 3.1.1. Micro-Mechanical Properties (Indentation Hardness, Indentation Modulus)

**Figure 3:**
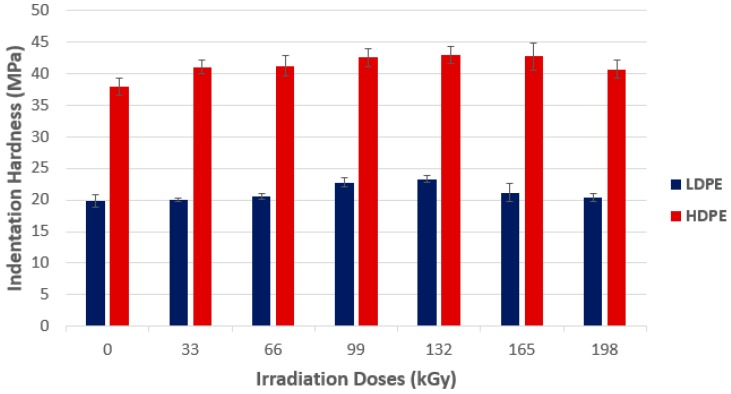
Indentation hardness (*H*_IT_)—Commodity polymers.

A graphic evaluation of the measured indentation hardness results in its dependence on different radiation doses for the selected commodity polymers (LDPE and HDPE), are shown in Figure 3. From the measured values of indentation hardness, it follows that radiation crosslinking of these commodity materials demonstrates an effect of higher hardness in the tested polymers. The lowest indentation hardness value was found in non-irradiated LDPE (19.9 MPa), and HDPE (37.9 MPa). The highest indentation hardness value was measured for both polymers irradiated at the dose of 132 kGy. The difference in hardness in the tested LDPE (23.3 MPa), was approximately 17%, while for HDPE (42.9 MPa), the difference was up to 14%. From Figure 3, it is clear that the indentation hardness of the tested commodity polymers are influenced by beta radiation. The drop in hardness values after the application of radiation doses higher than 132 kGy is probably caused by material degradation after irradiation. Statistical evaluation was carried out using the *T*-Test. Null hypotheses, and subsequent alternative hypotheses, certify the significant effect of radiation crosslinking on the tested polymer properties.

**Figure 4:**
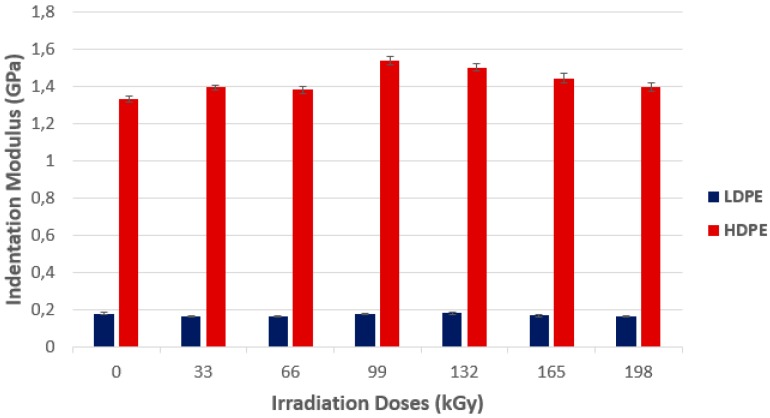
Indentation modulus (*E*_IT_)—Commodity polymers.

For the commodity polymer group, the changes of indentation modulus due to the irradiation of LDPE were meaningless (Figure 4). The HDPE indentation modulus increased with the radiation dose up to its maximum at the dose of 99 kGy (1.54 GPa), which is approximately higher by 16% in comparison with non-irradiated HDPE (1.33 GPa). The higher the radiation dose, the lower the values of the indentation modulus.

#### 3.1.2. Structural Properties

**Figure 5:**
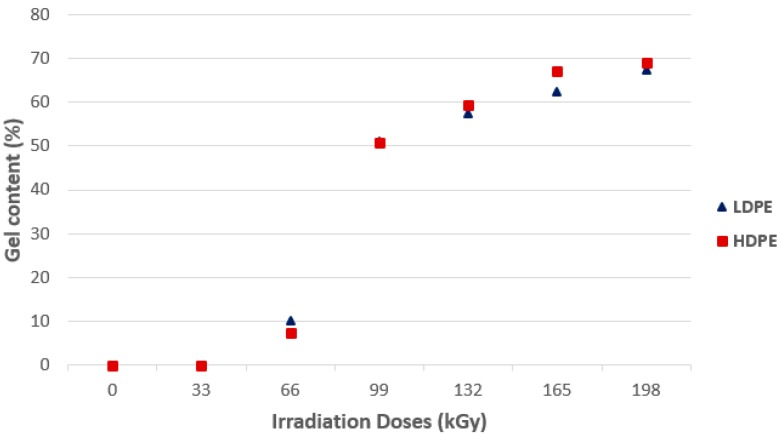
Gel content—Commodity polymers.

The gel-test is carried out for the purpose of measuring non-filterable phase content—The gel of the given material accords to the EN ISO 579 standard. Determination of gel content for LDPE and HDPE in dependence on the applied radiation dose are obvious from Figure 5. Connection lines between individual measurements do not express linear behavior, but only highlight the trend.

As is evident from Figure 5, the highest value of the gel content was achieved in both materials, LDPE and HDPE, at the highest radiation dose of 198 kGy. The LDPE and HDPE did not show significant increases of gel content up to the dose of 66 kGy. As was obvious from the mechanical and thermo-mechanical properties measurements, material irradiated at lower doses showed changes. The accuracy of the gel content measurement is largely dependent on the robustness of the filter screen used; and so, the resulting “micro gels” of the material in the lower radiation dose range can pass through the filter sieve and, so, they are not counted in the total gel content. However, they already play a significant role in the measured properties.

In order to confirm the results of, and changes to, the micro-mechanical properties of the polymer groups that were tested—As determined by the DSI method. Further measurements were performed, with the aim of describing the (concrete) changes in the structure and morphology of these polymers, irradiated by beta radiation tests. One material from each group was chosen. A deeper analysis of its structural properties was conducted.

**Figure 6:**
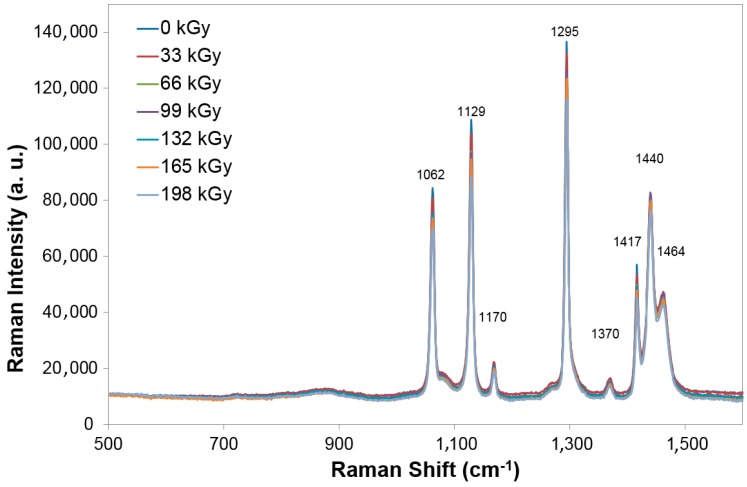
Raman spectroscopy—Commodity polymers (HDPE).

As regards the HDPE samples, Raman spectroscopy showed only small changes in structure, in comparison with non-irradiated and irradiated samples irradiated by different doses of radiation (Figure 6).

**Figure 7:**
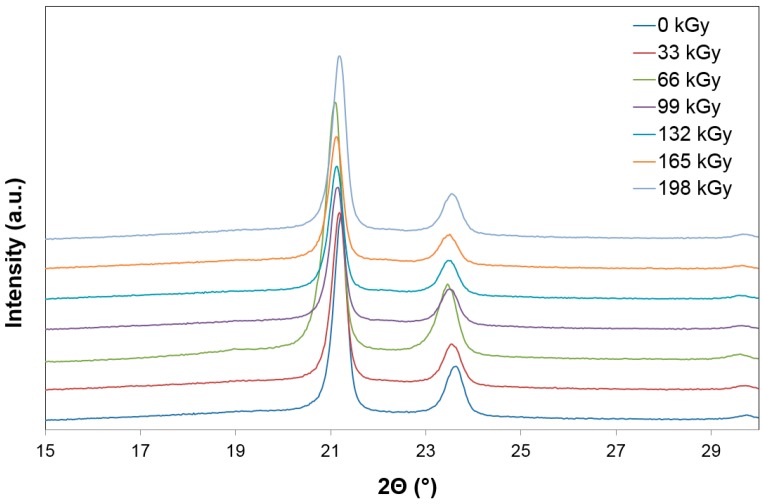
X-ray diffraction—Commodity polymers (HDPE).

**Figure 8:**
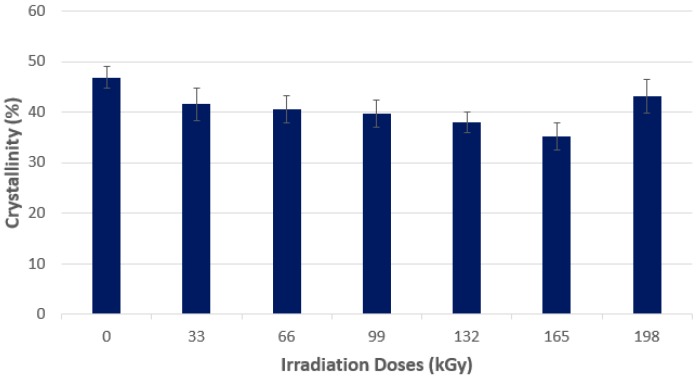
X-ray diffraction—Crystallinity—Commodity polymers (HDPE).

The HDPE tested by using wide-angle X-ray diffraction discovered that the highest amount of the crystalline phase was in the non-irradiated HDPE sample, which, simultaneously, showed the lowest micro-hardness *H*_IT_ values and the indentation modulus *E*_IT_ of the surface layer. With every increase of the radiation dose, the drop of the crystalline phase amount was measured. The lowest values were found at a dose of 165 kGy, as is visible from Figure 7 and Figure 8. The crystallinity results confirm these changes of the measured properties (indentation hardness and indentation modulus). The results that were obtained were close to the changes in indentation hardness *H*_IT_, and indentation modulus *E*_IT_ (Figure 3 and Figure 4).

For polyethylene, the increasing radiation dose leads to increases as the crystalline phase decreases, which is due to the fact that parts that were crystalline in the non-irradiated sample transform into its amorphous phase. At the radiation dose of 198 kGy, irradiation is affected by chain irradiation in the amorphous phase—Which causes the loosening of the chains able to associate with other strings, thereby increasing the crystalline phase.

#### 3.1.3. Thermo-Mechanical Properties

Temperature stability is assessed by using thermo-mechanical analysis, which is a precise evaluation technique of temperature resistance against hot probes, with a loading of 160 mN. The sensitive probe records data in a set range of temperatures; measurement times make it possible to observe graphical depictions. The second form of temperature stability evaluation is visual observation within the temperature chamber. It is not so precise, nor as sensitive, a technique as TMA but, from the macroscopic point of view, deformation under temperature is clear at first sight.

Commodity polymers, like LDPE and HDPE, usually demonstrate poor temperature stability, but are frequently used, since the preparation of products from these materials is simple and well-known. In addition, their chemical resistance is excellent and, therefore, material modification is an especially promising way of improving temperature stability. Sometimes, modified commodity materials can be considered to be technical materials in some fields and their properties—For example, radiation crosslinked PP with a 30% content of glass fiber.

**Figure 9:**
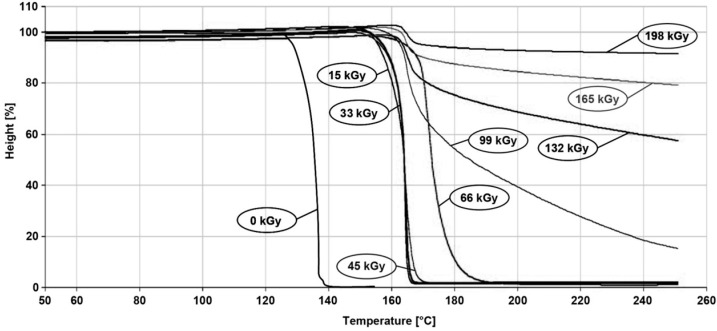
TMA—Commodity polymers (HDPE).

For the thermo-mechanical analysis of commodity polymers and their visual observation; certain representatives of HDPE commodity polymers were chosen, and their thermo-mechanical properties were measured. The thermo-mechanical properties were evaluated by TMA measurement in penetration mode. The graphical depiction of the TMA results describe the radiation dose, from 0 to 198 kGy. The irradiation effects of the thermo-mechanical properties of the HDPE studied, and are shown in Figure 9. The non-irradiated sample was melted at a temperature of 135 °C. The irradiated HDPE samples, with a small irradiation dose of 33, softens at a temperature of 165 °C. In connection with the increasing irradiation dose, temperature stability increases, and the irradiated HDPE specimen—At the highest irradiation dose used—Evinced the significant improvement of temperature stability.

**Figure 10:**
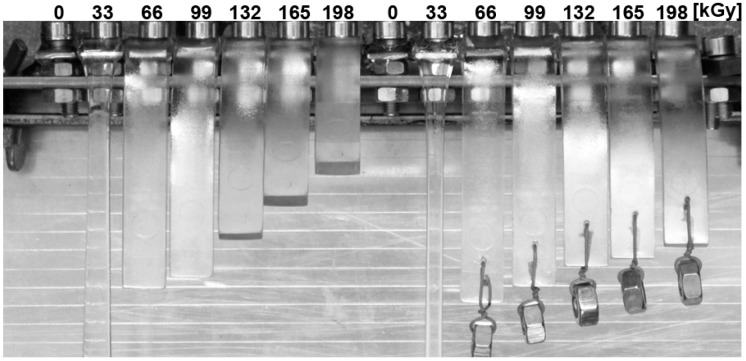
Visual observation after 1 h at 180 °C—Commodity polymers (HDPE).

The second temperature stability evaluation technique used the visual observation of deformed HDPE samples under temperature. The visual observation of sample behavior, after a one-hour exposition, at 180 °C, is given in Figure 10. The specimens are fitted horizontally in the temperature chamber, and loaded by the bending moment both of its own weight and the weight on the end of the specimen. Specimen deformation decreases in line with the increasing radiation dose at an elevated temperature. At 180 °C, the non-irradiated HDPE specimen is totally melted, while the same polymer, irradiated by the dose of 198 kGy, retains its cross-section without changes; there is only some deformation due to—And by—Its own weight. Visual observation makes it possible to see a similar tendency, as seen from the TMA analysis results.

### 3.2. Technical (Construction) Polymers

#### 3.2.1. Micro-Mechanical Properties—(Indentation Hardness, Indentation Modulus)

Similar properties to the selected commodity polymers were also measured for technical polymers by means of the DSI method. Radiation crosslinking also takes effect by increases in the indentation hardness of these polymers.

**Figure 11:**
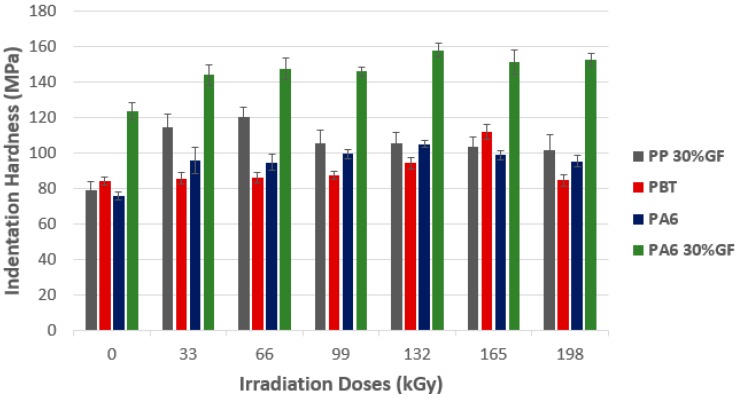
Indentation hardness (*H*_IT_)—Technical polymers.

The lowest indentation hardness values measured for all non-irradiated technical polymers were PP 30% GF (79.2 MPa), PBT (84.4 MPa), PA6 (76.1 MPa), and PA6 30% GF (123.7 MPa). For filled polypropylene, the highest indentation hardness values were found at lower radiation doses (33 and 66 kGy), where the difference between 0 and 66 kGy (120.1 MPa) was 51%. As for PBT, indentation hardness increased proportionately to the radiation dose, with the maximum dose of 165 kGy (111.9 MPa), where the difference between irradiated and non-irradiated material was 34%. The increases in radiation doses do not lead to the increase of indentation hardness; on the contrary, at the dose of 198 kGy, a significant decrease of indentation hardness, up to the non-irradiated polymer values, was measured. From a closer point-of-view on the indentation hardness results of PA6 and PA6 30% GF, it is clear that, at the radiation dose of 132 kGy, measurements found that this was the highest indentation hardness values. The difference, determined for PA6 (105.1 MPa), was 28%, while, for PA6 30% GF (157.9 MPa), this difference increased to the value of 34%. As is evident from Figure 11, as regards commodity materials, the highest indentation hardness values, using the DSI method, were measured at higher radiation doses, when the optimal dose always depends on the polymer type. Higher radiation doses do not significantly influence the indentation hardness value. Only slight changes occur.

**Figure 12:**
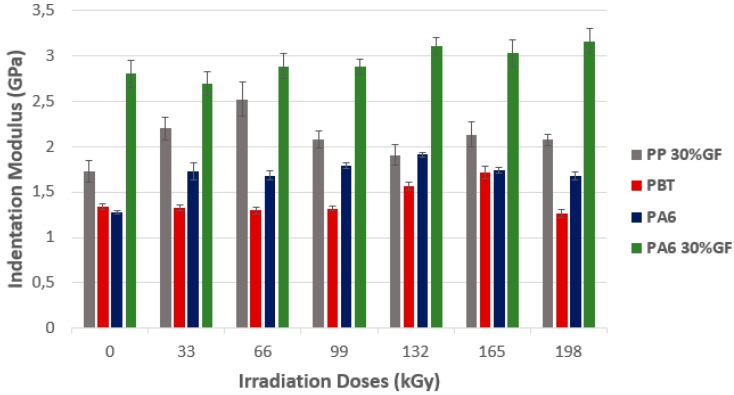
Indentation modulus (*E*_IT_)—Technical polymers.

Technical polymers showed an increase of the *E*_IT_ indentation modulus, in line with the radiation dose (Figure 12). For PP 30% GF, the maximum was reached at the dose of 66 kGy, with the difference of approximately 42% in comparison with non-irradiated material. With higher radiation doses, a significant drop (about 20%) occurs with stagnation of this value at higher doses. In the course of PBT radiation crosslinking, the *E*_IT_ value remained without change during increases of radiation doses up to 99 kGy. The maximal value was reached at 132 kGy, with the increase of the *E*_IT_ value by 30% in comparison with a non-irradiated polymer. The higher the radiation dose, the lower the *E*_IT_ value. At the maximal dose of 198 kGy, the *E*_IT_ value dropped to the value of a non-irradiated polymer. The PA6 *E*_IT_ value increased gradually with regard to the higher radiation dose, where the maximum dose was 132 kGy. The *E*_IT_ increase was about 52% as compared to non-irradiated PA6. The higher the radiation dose, the lower the *E*_IT_ values. PA6 30% GF showed a slight increase of *E*_IT_ values, where the maximum dose was 198 kGy. The difference between the maximal measured value and the *E*_IT_ value measured at non-irradiated PA6 30% GF, was up to 15%.

#### 3.2.2. Structural Properties

**Figure 13:**
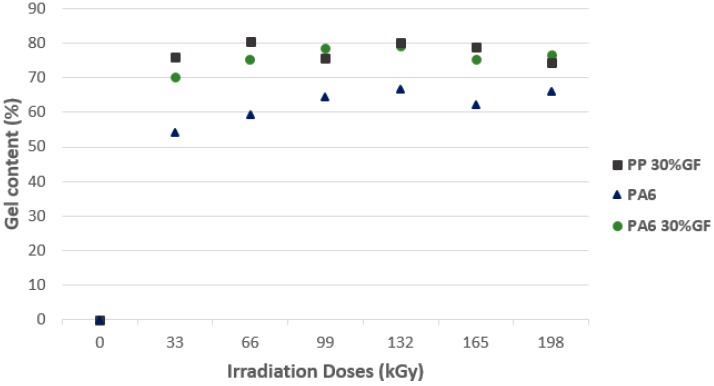
Gel content—Technical polymers.

Figure 13 depicts the results of the gel test on the following structural materials (PP 30% GF, PA6, PA6 30% GF). The non-irradiated test sample completely dissolves, confirming the results of the previous tests where the lowest micro-mechanical and thermo-mechanical properties values were measured. Significant gel growth was observed at the lowest radiation dose of 33 kGy, with increasing radiation doses, the gel content of all tested structural materials increased slightly. For PP, the maximal gel value was measured at a radiation dose of 66 kGy, which corresponds to the results of the micro-mechanical properties; where the maximal indentation hardness value and the indentation modulus were measured. For PA6-filled and non-filled polypropylene, the maximal gel value was measured at the radiation dose of 132 kGy. At this dose, the maximal micro-mechanical property values were also measured. At higher radiation doses, a slight decrease of the gel was measured, which corresponds to the micro-mechanical and thermo-mechanical properties results.

**Figure 14:**
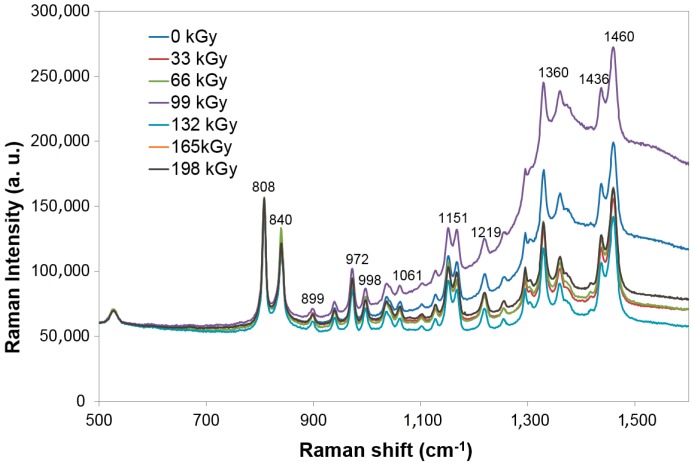
Raman spectroscopy—Technical polymers (PP 30% GF).

Raman spectroscopy of the analyzed filled PP samples does not show significant changes in the dependence on the radiation dose in comparison with wide-angle X-ray diffraction (Figure 14).

**Figure 15:**
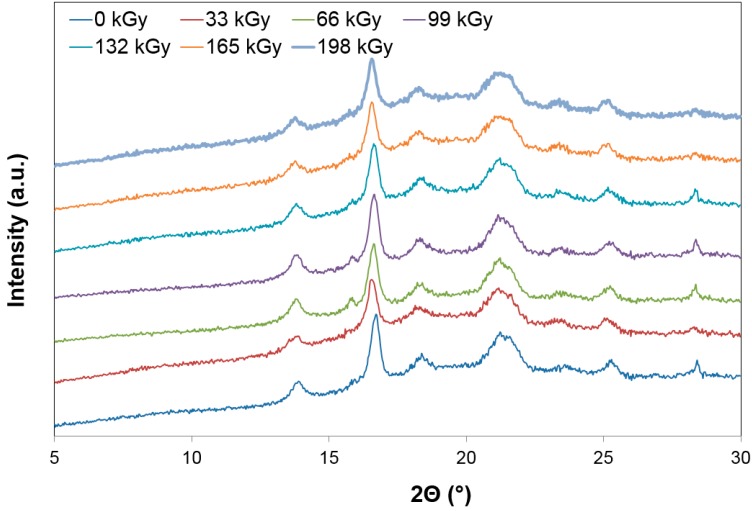
X-ray diffraction—Technical polymers (PP 30% GF).

**Figure 16:**
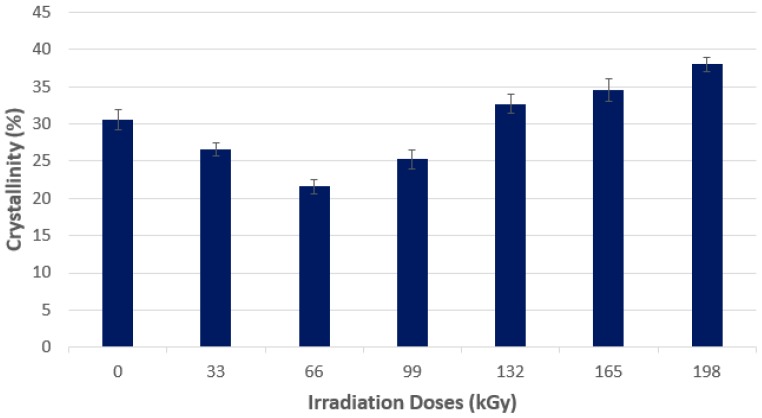
X-ray diffraction—Crystallinity—Technical polymers (PP 30% GF).

As is visible in Figure 15 and Figure 16, using wide-angle X-ray diffraction at PP 30% GF, the lowest amount of the crystalline phase was measured at the dose of 66 kGy, which simultaneously showed the highest micro-hardness and indentation modulus of the surface layer values. The highest values of the crystalline phase of the tested PP 30% GF were found at the dose-level of 198 kGy. In polypropylene, two processes occur at the same time—Radiation (degradation) and crosslinking. The use of a crosslinking agent leads to the crosslinking process prevailing, at the expense of degradation. At radiation doses higher than 66 kGy, the proportion of the crystalline phase is increased, mainly due to the cleavage of the amorphous phase of the polymer.

#### 3.2.3. Thermo-Mechanical Properties

Technical polymers, like PBT and PA6, can work on a permanent basis—Up to 120 °C—Depending on the kind of plastic material. They perform their function under normal conditions, just as classical materials do; however, at higher temperatures, technical polymers lose their properties. These materials are often used in the automotive industry for interior and exterior applications. Fillers (glass fibers, for instance) are used due to their better mechanical and thermal properties. The use of modifications can also improve temperature stability; one such modification possibility is radiation crosslinking, which is used in this work.

**Figure 17:**
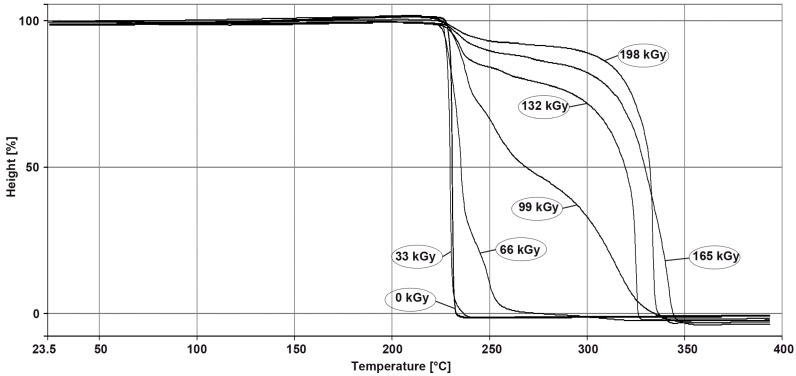
TMA—Technical polymers (PBT).

For thermo-mechanical analysis of technical polymers and visual observation, PBT was chosen as a representative example. Its thermo-mechanical properties were evaluated by means of TMA measurement, in penetration mode, on this material (Figure 17). From the TMA record, it can be seen that the non-irradiated sample melted at 230 °C; in addition, the same temperature softens the irradiated PBT sample with an irradiation dose of 33 kGy. Then, the increased irradiation dose led to the rise in temperature stability. Irradiated samples with doses more than 132 kGy can withstand 300 °C over a short time, without fatal damages to the product.

**Figure 18:**
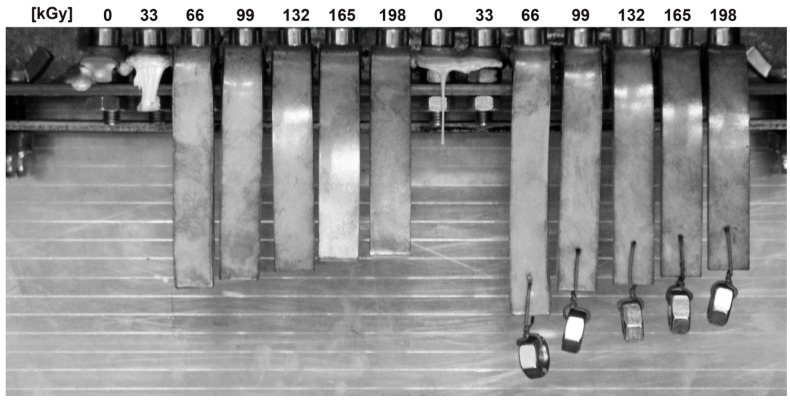
Visual observation after 1 h at 250 °C—Technical polymers (PBT).

A visual depiction of sample behavior, after a one-hour exposition at 250 °C, is given in Figure 18. At 250 °C, the specimens from non-irradiated PBT and the irradiated sample with the irradiation dose of 33 kGy are totally melted, while the polymer irradiated by doses from 99 to 198 kGy keeps its cross-section without changes. There is only deformation, due to its own weight. Additionally, visual observation of the PBT confirms the trends from the TMA analysis.

### 3.3. High-Performance Polymers

#### 3.3.1. Micro-Mechanical Properties (Indentation Hardness, Indentation Modulus)

**Figure 19:**
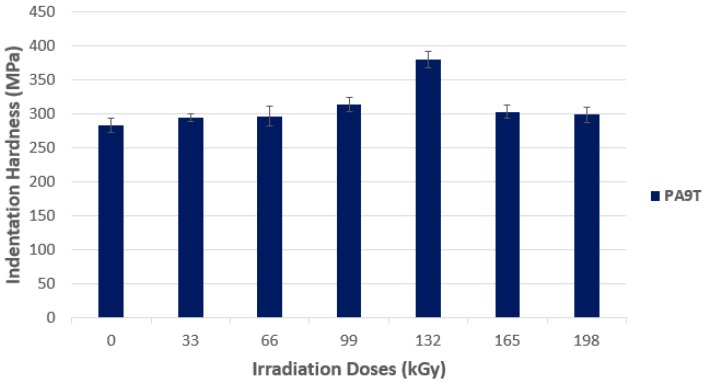
Indentation hardness (*H*_IT_)—High-performance polymers.

Radiation crosslinking of high-performance polymers, like PA9T, manifests itself by significant increases in hardness values, as is visible in Figure 19. The lowest indentation hardness values were measured for non-irradiated PA9T (283.3 MPa). On the contrary, the highest values were achieved by PA9T, irradiated by the dose of 132 kGy (379.7 MPa). The difference in hardness for both samples was approximately 34%. The hardness increase is caused by crosslinking, as a result of irradiation of the test body. The drop of both values after the application of radiation doses higher than 132 kGy, is probably caused by material degradation after irradiation.

**Figure 20:**
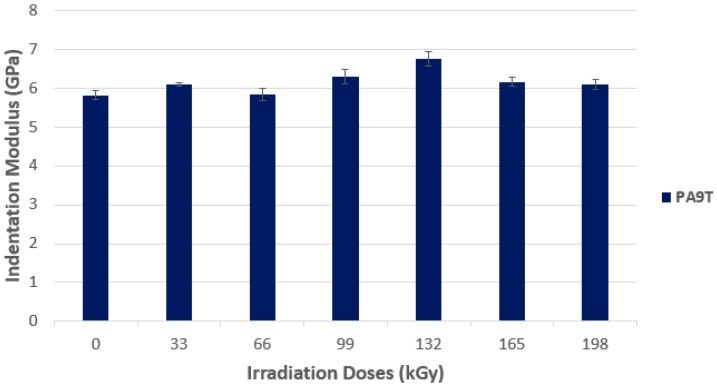
Indentation modulus (*E*_IT_)—High-performance polymers.

The high-performance polymer PA9T showed the increase of the indentation modulus *E*_IT_, in line with the increasing radiation dosage (Figure 20). For PA9T, the maximal value was measured at the dose of 132 kGy (6.7 GPa); with the increase of the *E*_IT_ value by 17%, in comparison with non-irradiated PA9T. The higher the radiation dose, the lower the *E*_IT_ value. The lowest value of indentation modulus was found among the non-irradiated materials (5.8 GPa).

As is apparent from the results of the measurement of indentation hardness and indentation modulus, during which essential changes of the mechanical properties of the selected material types occur due to the application of different beta radiation doses. Thanks to the differentiated application of radiation doses, improvements in the usual material properties occur; thereby, cheaper commodities or technical materials can replace more expensive technical or high-performance polymers. These results will be studied and confirmed by means of structural and morphological measurements.

#### 3.3.2. Structural Properties

**Figure 21:**
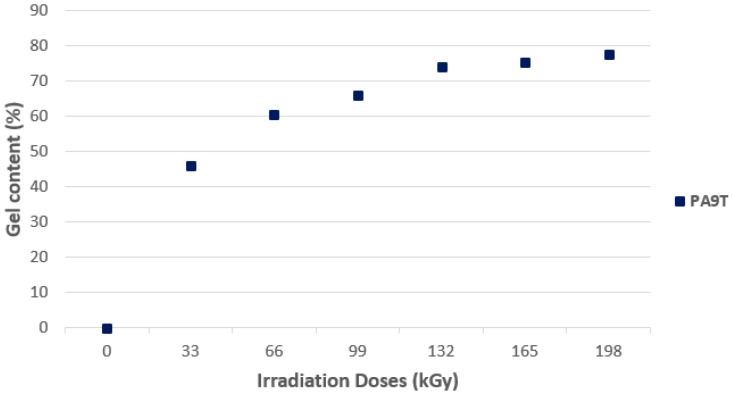
Gel content—High-performance polymers.

Figure 21 shows the resultant content of the PA9T gel. The trend in this material is similar to that of the PA6 trend; there is also a significant increase in the gel content at a radiation dose of 132 kGy (74%) and, with increasing radiation doses, the gel content increases slightly, up to a maximum radiation dose of 198 kGy, where the gel content was measured at 78%. These results correspond to the micro-mechanical properties measurement, where the highest values measured were at the radiation dose of 132 kGy, as well as the results of the thermo-mechanical analysis, where the best results were measured at the maximal radiation dose.

#### 3.3.3. Thermo-Mechanical Properties

High-performance polymers, like PA9T, can work under specific conditions, especially at higher temperatures, (at 200 °C and higher), on a permanent basis with the same properties as under normal conditions. These materials are used in special applications; for example, car and aerospace industry engine space parts. High-performance polymers are usually highly reinforced (50–60% glass fiber, or other fillers), so these high-tech materials are already modified by some improved process, not only glass fiber filling. Radiation crosslinking improves, above all, temperature stability, but not as considerably as for commodity and technical polymers.

**Figure 22:**
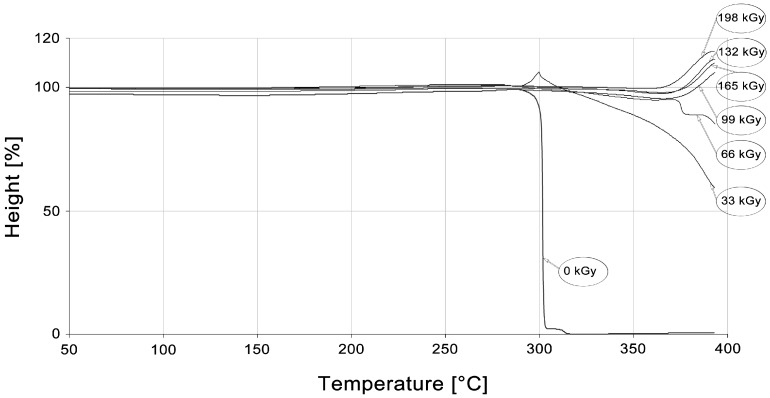
TMA—High-performance polymers (PA9T).

For thermo-mechanical analysis of high-performance polymers and their visual observation, PA9T was chosen as a representative. Its thermo-mechanical properties were evaluated by means of TMA measurement, in penetration mode, on this material. From the TMA record, it can be seen that the non-irradiated sample melted rapidly at the temperature of 300 °C, as is displayed in Figure 22. Temperature stability increased after the irradiation of the PA9T samples; however, there are small changes in the recorded data. From Figure 22, it follows that it is possible to use the PA9T polymer at surrounding temperatures much higher than the melting point of the virgin (non-irradiated) polymer. The products made from PA9T, crosslinked by the irradiation dose of 66 kGy, could work at the temperature of 350 °C during a certain time without fatal damage.

Figure 22 shows the thermal behavior of PA9T at elevated temperatures. From this figure, it follows that it is possible to use this type of polymer in surrounding temperatures higher than the melting point of virgin (non-irradiated) PA9T. The unusual course of the TMA curve of the polymer irradiated by the dose higher than 99 kGy is caused by the inflation of the specimen due to gas evolving at temperatures higher than 370 °C (probably due to the decomposition of the unused remnants of the crosslinking agent).

**Figure 23:**
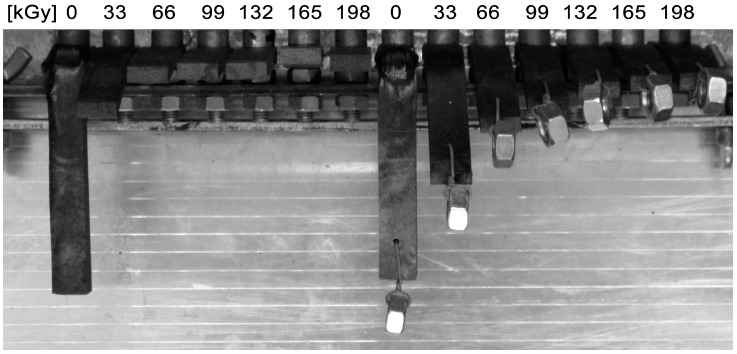
Visual observation after 1 h at 300 °C—High-performance polymers (PA9T).

The visual observation of sample behavior after a one-hour exposition at 300 °C is given in Figure 23. At 300 °C, the specimens from non-irradiated PA9T are deformed, while the polymer irradiated by doses from 33 to 198 kGy, retains its cross-section without changes; there is only some deformation due to its own weight. Additionally, PA9T visual observation confirms the trends from the TMA analysis. However, this material changes color from natural to black; there is evident degradation by thermo-oxidative processes.

## 4. Conclusions

From the above results, it is evident that beta radiation crosslinking has an influence on the micro-mechanical and thermo-mechanical properties of selected types of polymers. The polymers were selected across the whole area of thermoplastics-covering commodity polymers (LDPE, HDPE), technical polymers (PP 30% GF, PBT, PA6, PA6 30% GF) and, also, high-performance polymers (PA9T). These polymers were modified using beta radiation of differing radiation doses (33, 66, 99, 132, 165, and 198 kGy).

The micro-mechanical properties (indentation hardness and indentation modulus), were measured using the DSI method for all of the polymers tested. LDPE and HDPE irradiated at the dose of 132 kGy (17% and 14%); polypropylene irradiated at the dose of 66 kGy (51%); PBT irradiated at the dose of 165 kGy (34%); and polyamides irradiated at the dose of 132 kGy (PA6, 38%; PA6, 30%; GF, 28%; and PA9T, 34%) reached the highest levels of changes of the tested properties in comparison with non-irradiated materials. Nevertheless, it is necessary to mention that it is always necessary to thoroughly consider the final radiation dose with respect to the efficiency of the irradiation process. In some cases, it is possible to also choose a lower radiation dose with comparable modification results being reached.

The thermo-mechanical analysis confirmed the influence of radiation doses on the structure of the polymers that were studied. The higher the dose of radiation, the higher the thermal resistance of the studied polymers above the values of melting temperatures of a basic polymer, which was compared with the knowledge gained from the literature. Polymers change their behavior from thermo-plastic to thermo-elastic after radiation crosslinking. From the measurements, it can be stated that the highest changes were achieved by all materials examined at higher doses of radiation.

The micro-mechanical properties and thermo-mechanical properties results were supported by the measurement of the structure, where gel test measurement, wide-angle X-ray diffraction, and Raman spectroscopy tests were performed.

It should also be noted that a higher dose of radiation intensity does not necessarily mean a higher improvement in the desired properties. For a particular application and material it is always necessary to look for a suitable radiation dose intensity.

## Figures and Tables

**Table 1:** Tested polymers.

Type of Polymers	Trade Name	Company
Low density polyethylene	DOW LDPE 780 E	DOW (Midland, MI, USA)
High density polyethylene	DOW HDPE 25055 E	DOW (Midland, MI, USA)
Polypropylene with 30% of glass fibers	PP V-PTS-CREALEN-EP8G5HS * M0083	PTS (Adelshofen, Germany)
Polybuthylene terephthalate	PBT V-PTS-CREATEC-B3HZC * M800/25	PTS (Adelshofen, Germany)
Polyamide 6	V-PTS-CREAMID-B3H2 * M800/14 natur	PTS (Adelshofen, Germany)
Polyamide 6 with 30% of glass fibers	PA 6 FRIANYL B63 VN GV 30 schwarz 9005	FRISETTA (Utzenfeld, Germany)
Polyamide 9T	V-PTS-DURAMID-9TH2G9 * M800/13 natur	PTS (Adelshofen, Germany)

**Table 2:** Process parameters.

	LDPE	HDPE	PP 30% GF	PBT	PA6	PA6 30% GF	PA9T
Injection velocity (mm/s)	50	60	100	80	50	50	50
Injection pressure (MPa)	60	80	80	80	65	65	170
Cooling time (s)	30	20	50	35	17	17	25
Mold temperature (°C)	40	40	45	85	70	70	140
Holding time (s)	30	25	15	25	20	20	15
Zone 1 (°C)	190	200	210	210	220	220	295
Zone 2 (°C)	200	205	220	220	250	250	305
Zone 3 (°C)	210	210	230	235	270	270	315
Zone 4 (°C)	215	225	240	250	280	280	325
